# A Novel Metamaterial Inspired High-Temperature Microwave Sensor in Harsh Environments

**DOI:** 10.3390/s18092879

**Published:** 2018-08-31

**Authors:** Fengxiang Lu, Qiulin Tan, Yaohui Ji, Qianqian Guo, Yanjie Guo, Jijun Xiong

**Affiliations:** 1 Key Laboratory of Instrumentation Science & Dynamic Measurement, Ministry of Education, North University of China, Taiyuan 030051, China; peaceliveluna@163.com (F.L.); jyhzbdx@163.com (Y.J.); qqguo0214@163.com (Q.G.); 18235140097@163.com (Y.G.); xiongjijun@nuc.edu.cn (J.X.); 2 Science and Technology on Electronic Test and Measurement Laboratory, North University of China, Taiyuan 030051, China

**Keywords:** SRR-based sensor, electromagnetic backscatter principle, chipless radio frequency identification, high-temperature sensor

## Abstract

A high-temperature sensor based on a metamaterial unit cell is proposed in this paper. The wireless passive temperature sensing method is based on the electromagnetic backscatter principle, and thus has the advantages of higher quality, lower environmental interference, and anti-low frequency interference. We developed a finite-element method-based model for the sensor via high-frequency simulation software (HFSS). A double split-ring resonator (SRR) with an outer ring length of 13 mm was designed on alumina ceramic substrate. The sensor was fabricated at 2.42 GHz using micromechanical technology and screen printing technology. When the temperature increased from 28 to 1100 °C, the resonant frequency decreased from 2.417 to 2.320 GHz with an average sensitivity of 95.63 kHz/°C. As the sensor is easily designed and fabricated, it can be used for chipless radio frequency identification (RFID) tags by simply changing the size of rings. Furthermore, emerging 3D printing technology and commercial desktop inkjet printers will be used to realize the rapid low-cost preparation of the sensor, enabling its wide range of applications in aerospace, military, manufacturing, transportation, and other fields.

## 1. Introduction

High-temperature sensing is in great demand in the aerospace and manufacturing industries. Real-time sensing and in situ temperature monitoring are critical in improving the working efficiency of devices and ensuring the safe operation of equipment. For example, the excessively high temperature of a furnace wall or pipe wall can lead to its deformation or bursting, which will reduce work efficiency and cause safety risks [1,2]. In a combustion chamber, the combustion temperature directly affects the engine’s working efficiency and output power [3,4]. When the blade of a turbine engine runs at a high speed, an increase in the surface temperature causes the blade to deform [5,6]. Therefore, the applications of high-temperature sensors that are stable and function in temperatures up to 1000 °C are extensive. Such sensors are required to be small, easy to manufacture, inexpensive, and highly reliable.

Microwave scattering sensing technology has become increasingly popular owing to its higher quality, lower environmental interference, and anti-low frequency interference compared to near-field coupled capacitive-inductive (LC) sensing technology and surface acoustic wave (SAW) sensing technology. For instance, Cheng et al. [7] designed a microwave temperature sensor and tested it in temperatures from 50 to 1050 °C it showed a high sensitivity of 0.58 MHz/°C at 1050 °C. This sensor can be used as temperature sensor and antenna simultaneously and its characteristic of stability at high frequencies enables its survival in harsh environments. Xiong et al. [6] reported a sensor with a center frequency of 2.4 GHz which can work in temperatures from 27 to 800 °C with a sensitivity of 0.19 MHz/°C. This sensor is based on a microwave dielectric resonator and experiments have proved that it can perform effective monitoring at high temperatures. The recently developed chipless radio frequency identification (RFID) technology is based on the microwave backscatter principle. The tags can transmit information about its current temperature back to the reader via wireless transmission. It is widely used in environment parameter sensing, as it can simultaneously identify targets and detect information about the target object. The tags do not need to be powered by a battery [8], can be fabricated at a low cost, and have a long lifetime [9]. For example, Herrojo et al. [10] proposed a novel near-field chipless RFID system used for angular velocity measurements by operating in time domain and based on sequential bit reading. Vena et al. [11] fabricated a low-cost wireless fully inkjet-printed chipless sensor on a flexible laminate for CO_2_ detection. Temperature is a vital factor that must be monitored in harsh environments. Currently, there has been little research on high-temperature sensing using this method, hence the research of high-temperature sensors combined with chipless RFID technology is necessary and promising.

The potential applications of metamaterials have emerged because of their unique electromagnetic properties under the irradiation of electromagnetic waves at certain frequencies [12]. These metamaterials are periodically arranged from subwavelength, high-conductivity metal structures and are fabricated on a dielectric substrate. One of the most fundamental periodical unit cells is a split-ring resonator (SRR) [13]. Several studies have been reported that any geometric deviation caused by external strain applied to SRR devices and any sensitive materials attached to the devices’ surface result in a resonant frequency change of the resonator [13,14,15]. SRR-based sensors have been applied to various situations, such as medical monitoring (i.e., glaucoma monitoring) [13], strain measurement [14], biological detection [15], and early detection of breaches in pipeline coatings [16]. The substrate dielectric constant also has an effect on the resonant frequency of SRR-based sensors. Our previous research has shown that it is reliable and feasible to directly measure the temperature dependence of the dielectric constant of the substrate material [17,18,19]. Therefore, dielectric constant sensitive SRR-based sensors in combination with chipless RFID technology is predicted to be very suitable for microwave temperature sensing with advanced properties.

In this paper, we propose a novel approach for pushing the temperature limits of the devices by combining a metamaterial unit cell with high-temperature co-fired ceramic (HTCC) technology and chipless RFID technology. A thin-film sensor for high-temperature sensing based on the microwave backscattering principle has been fabricated. This sensor embeds two interleaved metallic rings with two opposite gaps on alumina ceramic, and is easily fabricated, has a flexible design, and has a high sensitivity. Experimental results show that the sensor can work up to 1100 °C with an average sensitivity of 95.63 kHz/°C around 2.42 GHz. The size of the SRR structure can be reduced to micro- and nanoscales, thus the miniaturization of these sensors can be achieved. In addition to measuring ultra-high temperatures in harsh environments, some temperature-sensitive nanomaterials can also be used to measure temperatures that are not so high. For example, graphene can be coated on the SRR structures for long-term passive temperature measurements around 150 °C. In this case, sensors can be printed on a flexible substrate with conductive ink, such as a paper and plastic film with a commercial desktop inkjet printer. This will allow SRR-based sensors further applicability to a variety of devices.

## 2. Materials and Methods

**Figure 1:**
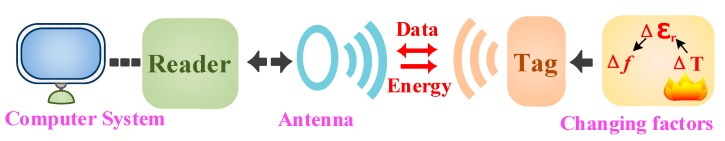
Principle of chipless radio frequency identification (RFID) systems for temperature sensing.

The principles of the chipless RFID system are shown in Figure 1. The system consists of three parts: tag, reader, and a computer data processing system. The chipless RFID tag relies entirely on the conductor printed on the tag to store information and uses a type of frequency-domain coding. A reader antenna is used to receive or transmit electromagnetic waves. When the tag is irradiated by an electromagnetic wave, the tag can be coupled with the electromagnetic wave through its own structure characteristics, and then the structure characteristic information is returned to the reader. Each tag will reflect signals of different characteristics in a backscattering manner and the reader can recognize the target by interpreting the structural information of the tag. The computer data processing system then processes the return signals. In this study, we printed a SRR structure on an alumina ceramic substrate to form the sensing tag by using screen printing technology. When the temperature of the environment changes, the dielectric constant of the alumina ceramic changes accordingly, which causes a sensitive and strong change in the capacitance of the narrow gap in the SRR structures and between the rings, resulting in a change of the resonant frequency of the tag. By using computer data software to process the return signal, the temperature change can be analyzed, and hence temperature sensing is achieved.

**Figure 2:**
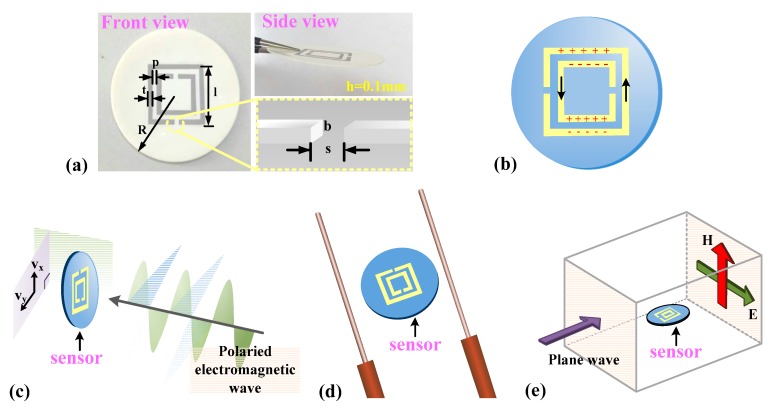
(**a**) Fabricated sensor displayed in front views. (**b**) Charge distribution in the metal rings when sensor irradiated in electromagnetic wave. Possible relative positions of the antenna and sensor (**c**) the sensor is irradiated by polaried electromagnetic wave (**d**) the sensor is placed between a pair of monopole antennae (**e**) the sensor is placed in vacuum waveguide.

The sensor embeds two interleaved metallic rings with two opposite gaps on alumina ceramic. The whole structure acts as an LC oscillator [20] in an external electromagnetic field causing sharp absorption of power corresponding to the resonance frequency. Figure 2a shows the schematic diagram and equivalent circuit diagram of the sensor. The resonant frequency (*f*_s_) is given as follows:

*f*_s_ = 1/(2π (*L*_s_ × *C*_s_)^−1/2^) (1)

Therefore, we mainly consider the influence of the changes in capacitance (*C*_s_) and inductance (*L*_s_) on the resonant frequency (*f*_s_). Ls related to the metal width t, the length of the outer ring sides *l*, the space between the inner and outer rings p, and the relative permeability of the dielectric matrix *μ*_r_ according to Reference [21]. The capacitance (*C*_s_) consists of two parts, the capacitance between the two rings (inter-ring capacitance *C*_i_) and the gap capacitance (*C*_g_), thus the whole capacitance can be expressed as follows [22]:

*C*_s_ = *C*_i_ + *C*_g_ (2)

*C*_s_ = 2.5∙*ε*_0_*ε*_r_∙(0.06 + 3.5 × 10^−5^ × (r_in_ + r_out_)) + 28∙*ε*_0_*ε*_r_∙A/g (3)

*ε*_r_ = *E*/*E*_e_ (4)

where *ε*_0_ and *ε*_r_ are the dielectric constant of vacuum and dielectric matrix, respectively, r_out_ and r_in_ represent the radius of the outer circumference and the inner circumference of the SRR, respectively, A is the cross-sectional area of the split of the metal ring. The relative dielectric constant (*ε*_r_) is usually defined as the ratio of the electric field in the absence of the dielectric (*ε*_0_) to the actual field intensity in the dielectric (*E*_e_) and can be expressed in Equation (4). The sensor can be irradiated by time varying electromagnetic wave which was generated by poled horn antenna or polarized patch antenna. Another way is to use a pair of monopole antennae or place the sensor in a vacuum waveguide to realize sensing function. When using the second method, it is necessary to ensure that the direction of the electric field should be perpendicular to the symmetrical plane of the rings. This is because there is an electric dipole in the direction perpendicular to the symmetry plane (electric wall) of the particle at the first resonant frequency when the sensor is irradiated in an electromagnetic wave, the charge distribution in the metal rings is depicted in Figure 2b. This simultaneously proves that the sensor can be irradiated by means of a time varying electric field applied in the plane of the particle codirectional to the direction of the electric dipole. The possible relative positions of the antenna and sensor are shown in Figure 2c–e.

When putting a sensor into an alternating electromagnetic field, alumina ceramic dielectrics can generate polarized charges. The thermal motion of ions goes intense when temperatures increase, the orderliness caused by the effect of the electric field becomes lower, resulting in a weaker polarization of charges. This will make the actual electric field (*E*_e_) intensity become smaller and the higher the temperature, the faster the frequency change (as can be seen in Figure 8). The relationship between *E*_e_, *ε*_r_, *C*_s_, *T,* and f can be clearly described as follows:

*T*↑→*ε*_r_↑→*E*_e_↓→*C*_s_↑→*f*_s_↓

It can be seen from the above descriptions that the temperature is inversely proportional to the resonant frequency (*f*_s_), which makes the sensor suitable for microwave wireless passive temperature sensing applications.

## 3. Design and Fabrication

### 3.1. Implementation of Chipless RFID

**Figure 3:**
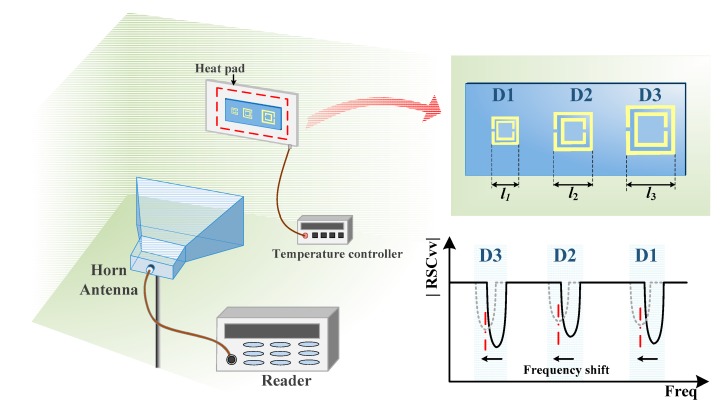
The schematic diagram of a chipless RFID system and tag coded with D1, D2, and D3.

From the above description, SRR structures can be used to easily fabricate ID tags because of their simple structures. The resonant frequency of each tag can be designed by changing the parameters of the SRRs and different IDs can be easily achieved by changing the length of the SRR rings. Figure 3 shows the schematic diagram of a chipless RFID system and a sketch of ID coded with D1 (*l*_1_), D2 (*l*_2_), and D3 (*l*_3_), respectively, (*l*_1_, *l*_2_, and *l*_3_ are the length of outer ring of each SRR, respectively). The longer the outer ring side length, the lower the resonant frequency. The tag is placed on the heat pad, the temperature of which is controlled by the temperature controller. A horn antenna is used for signal receive and transmit. When the external environment factors change, the resonance frequency of each ID can change in a certain range (the blue area in the lower right corner of Figure 3). This method can simultaneously identify targets and detect information about the environment.

### 3.2. Design and Fabrication of Temperature Sensor

**Figure 4:**
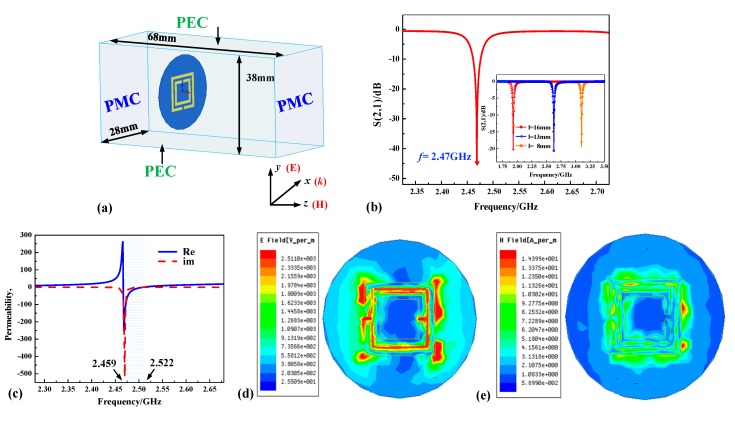
Simulation of the designed sensor. (**a**) Sensor modeling diagram via high-frequency simulation software (HFSS). (**b**) Transmission curve of the designed sensor. (**c**) The real and imaginary parts of the simulated magnetic permeability. (**d**) Electric field distribution of the sensor surface. (**e**) Magnetic field distribution of the sensor surface. PEC = perfect electric conductor; PMC = perfect magnetic conductor.

At present, 2.4 GHz band is one of the Industrial Scientific Medical (ISM) bands, thus obtaining wide application. Devices that work in the 2.4 GHz band can be used more widely and have a stronger anti-interference ability. Here, we choose one of the IDs in Figure 3 as a temperature sensor to achieve high temperature measurement. The SRR-based sensor was designed with a frequency of approximately 2.4 GHz (2.47 GHz). We used high-frequency simulation software (HFSS) to model and analyze the electromagnetic responses of the sensor. The sensor was placed in an S-band waveguide with dimensions of 28, 38, and 64 mm, as shown in Figure 4a. The simulation was performed by applying special boundaries to the waveguide. Perfect magnetic conductor (PMC) boundaries were set perpendicular to the *z* axes, while perfect electric conductor (PEC) boundaries and open boundaries corresponded to the *y* and *x* axes, respectively. The parameters of the designed sensor were finally determined as listed in Table 1. Figure 4b shows the simulation transmission curve of the proposed structure.

Permeability curves can be extracted from the S-parameter as shown in Figure 4c [23]. It can be clearly seen that the permeability (*μ*_r_) falls between 2.459 and 2.522 GHz, which indicates that the sensor can exhibit a negative permeability at certain frequencies. Therefore, the sensor is a μ-negative metamaterial. Figure 3d,e show the simulated electric field distribution and magnetic field distribution of the surface. The electric and magnetic fields have higher values between the two rings and gaps which is consistent with theoretical analysis.

**Figure 5:**
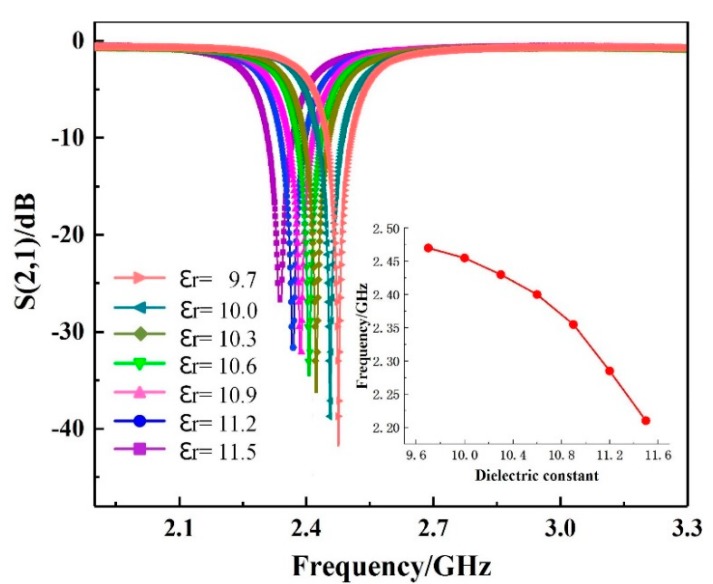
Frequency versus S (2, 1) for different dielectric constants of the alumina ceramic substrate. The insert chart is the frequency versus dielectric constant of the alumina ceramic substrate.

The sensor was realized using high-temperature co-fired ceramic (HTCC) technology, with the advantages of corrosion resistance and high thermal conductivity. It can also be fabricated using original 3D printing technology or directly sprayed on a device surface or furnace wall. We used 99% alumina with a dielectric constant of 9.7 at 28 °C, according to the data sheet provided by the producer. The performance index of alumina ceramics is summarized in Table 2. The material can withstand temperatures up to 1400 °C and has a low thermal expansion, high thermal conductivity, and high mechanical strength [21]. The high thermal conductivity can make the sensor accurately sense the ambient temperature with less error. Furthermore, the high mechanical strength makes the sensor hard to be damaged and can prolong the service life. When temperature increases from 28 °C to 1050 °C, the dielectric constant of the alumina substrate increases from 9.7 to 11.4 [7]. Figure 5 simulates the relationship between the resonant frequency and S (2, 1) with different dielectric constant values. We set the dielectric constants to be 9.7, 10.0, 10.3, 10.6, 10.9, 11.2, and 11.5; the simulation results (insert chart in Figure 5) show that the frequency monotone decreases from 2.47 to 2.21 GHz with the increase of the dielectric constant. Therefore, the use of alumina ceramic is suitable for high temperature measurements. Platinum conductor (U.S.A, ESL, 5541-A) was used as the metal in the resonant rings, as this can function in temperatures up to 1800 °C. The platinum paste was brushed onto the ceramic substrate using screen printing technology and then sintered in a high-temperature furnace. The sintering process consists of three stages: heating stage (180 min, 0 °C reaching to 1350 °C), insulation stage (stay at 1350 °C for 30 min), and cooling stages, respectively. By sintering at a high temperature, the organic material in the platinum conductor can be well combined with the ceramic matrix and eventually form a dense matrix structure.

## 4. Experiments and Discussion

**Figure 6:**
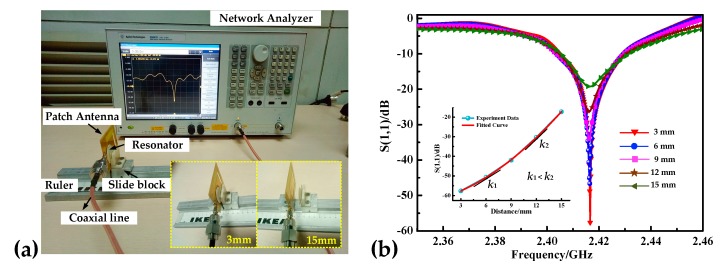
(**a**) Sensing distance testing platform. (**b**) Distance testing curves. The insert map indicates the relationship between S (1, 1) and distance and *k* is the slope of the fitting curve at a certain point.

We set up a distance testing platform (Figure 6a) to study the influence of the sensing distance on the return signal. The antenna used in the experiment was a patch antenna with bandwidth that contained the resonant frequency of the sensor. The reader antenna and sensor were mounted on a 2D stage machine and slider 1 remained stationary. By changing the distance (moving slider 2 to vary the interrogation distance) between the reader antenna and sensor, the resonant frequency of the sensor and impedance of the system can be changed. We used a network analyzer (Agilent E5061B) as the signal acquisition instrument and changed the distance from 3 to 15 mm in 3 mm intervals. It can be seen from the curves that the resonant frequency remains nearly unchanged while S (1, 1) decreases with an increase in distance. When the interrogation distance becomes small, most of the electromagnetic waves emitted from the antenna can cause the resonance of the sensor, and there is little electromagnetic wave attenuated in the free space, so a large return loss | S (1, 1) | can be obtained. The inset of Figure 6b shows the relationship between | S (1, 1) | and the interrogation distance (d). *k* is physically defined as attenuation rate of the impedance. *k*_1_ and *k*_2_ represent the slopes of the fitted curve at the interrogation distances of 6 mm and 12 mm. It can be seen that the return loss | S (1, 1) | increases with the distance and the attenuation is getting faster and faster.

The attenuation rate of the impedance (*k*) increases with an increase in distance (*k*_1_ < *k*_2_). When the sensing distance is larger than 20 mm, the return signal becomes indistinct. The value of | S (1, 1) | is about 60 dB and 20 dB when the query distance was 3 mm and 15 mm, respectively. The thickness of the furnace wall was approximately 10 to 20 mm. When measuring the temperature of the furnace wall, the sensor can be placed inside the wall with the antenna outside the furnace wall. In order to obtain strong signals, we set the distance between the reader antenna and the sensor to be 5 mm in later experiments. Due to the influence of the aluminum alloy components on the 2D working stage, the center frequency has a degree of deviation from the actual testing frequency, where there is no metal environment.

**Figure 7:**
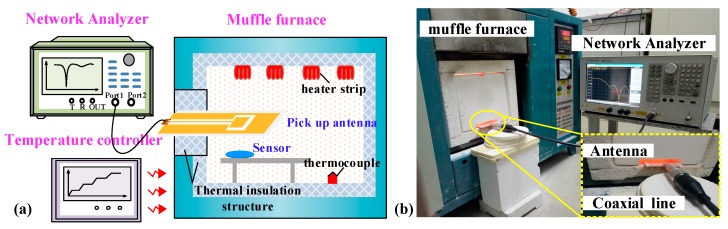
(**a**) Schematic of the high-temperature testing platform. (**b**) Measurement set up.

**Figure 8:**
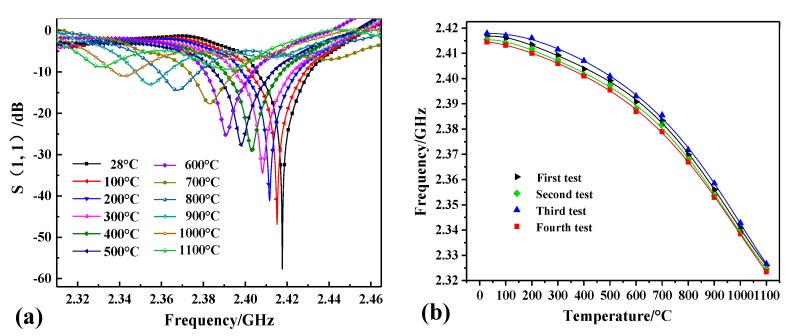
(**a**) S (1, 1) spectra of the sensor when the temperature is increased from 28 to 1100 °C; (**b**) Repeatability testing of the sensor.

To measure high temperatures, the fabricated sensor was placed in a muffle furnace. The schematic of the high-temperature test platform is shown in Figure 7. A mullite insulation layer was used to maintain the temperature inside the furnace. A coplanar waveguide (CPW) antenna with a center frequency of 2.02 GHz was used for wireless inquiry, with a relative bandwidth of 49.5% and maximum vertical gain of 1.7 dB. The reader antenna excites the sensor through the coaxial cable connected to the network analyzer (Agilent E5061B) and receives a return signal from the sensor. The temperature curve set by the temperature controller is sent to the high-temperature muffle furnace (Nabertherm LHT 02/1). The heating rate was set to 10 °C/min and dataset was recorded every 50 °C until | S (1, 1) | was less than 10 dB, which occurred when the temperature reached 1100 °C. The test curve is shown in Figure 8a. It can be seen from the test curve that the frequency of the sensor is 2.42 GHz and the value of | S (1, 1) | is −57.65 dB at 28 °C. The measured resonant frequency at 28 °C has slight deviation with the simulated resonant frequency, which may be caused by the inaccuracy of the fabrication process and relative constant of alumina ceramic. As the temperature increases from 28 to 1100 °C, the resonance frequency of the sensor is reduced from 2.417 to 2.320 GHz accordingly.

**Figure 9:**
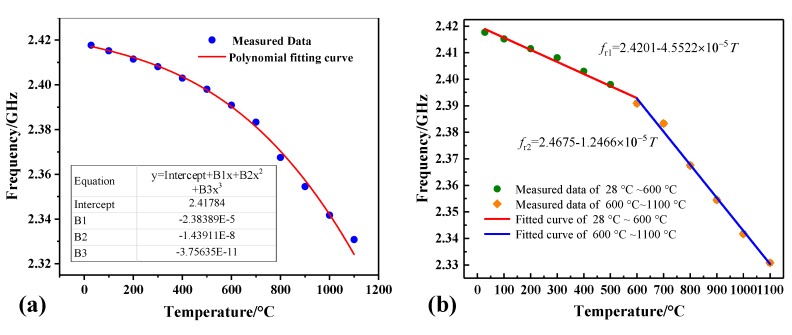
(**a**) Cubic fitting curve of average value of test data; (**b**) Relationship between temperature and resonant frequency polynomial fitting curves when temperature ranges from 28 to 600 °C, and 600 to 1100 °C.

Repeated experiments have been conducted to verify the feasibility of the sensor. Tests were repeated four times, and by extracting the frequency peak, the good repeatability of the sensor was verified in Figure 8b. We used the average value of four test experiments to make a frequency temperature curve. The relationship between temperature and frequency can be better obtained by a cubic polynomial, *f*_r_ means the frequency at a certain temperature (*T*) on the fitting curve and the fitting curve is expressed as follows (Figure 9a):

*f*_r_ = 2.42 − 2.38 × 10^−5^*T* + 1.44 × 10^−9^*T*^2^ − 3.76 × 10^−11^*T*^3^ (5)

We define S = ∆*f*_r_/∆*T* (kHz/°C) as the average absolute sensitivity of the proposed sensor, *k*’ = d*f*_r_/d*t* (kHz/°C) as the rate of change of frequency with temperature. The experimental results show that the average sensitivity of the sensor is 95.63 kHz/°C in the temperature range of 28–1100 °C and the value of | *k*’ | increases with the increase of temperature according to the principle mentioned in Section 2.

**Figure 10:**
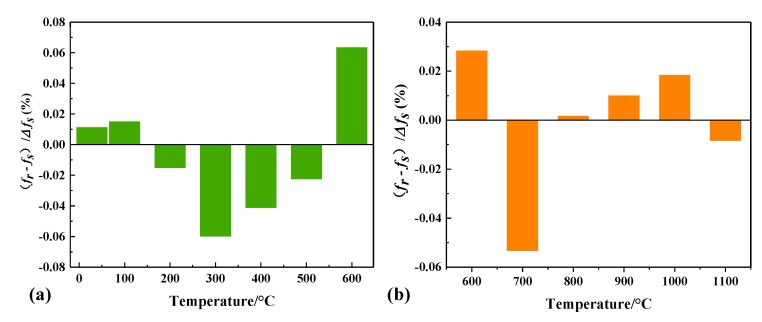
(**a**) Nonlinearity errors in the temperature range of 28 to 600 °C. (**b**) Nonlinearity errors in the temperature range of 600 to 1100 °C.

To simplify the expression of the sensor and make the sensor have excellent linearity in a certain temperature range, we divided the test results into two temperature ranges and performed a piecewise fitting of the test data (Figure 9b). The sensitivity of the sensor is found to be 45.5 kHz/°C in the range of 28–600 °C, 124.6 kHz/°C in the range of 600–1100 °C, respectively. In Figure 10, the SRR-based sensor had a maximum of 0.0017 GHz nonlinearity errors in the range of 28–600 °C, corresponding to 6.34% nonlinearity error; a maximum of 0.0032 GHz nonlinearity errors in the range of 600–1100 °C, corresponding to 5.32% nonlinearity error. Therefore, better linearity can be obtained by using different formulas in different temperature ranges:

*f*_r1_ = 2.4201 − 4.5522 × 10^−5^*T* (28 °C < *T* < 600 °C) (6)

*f*_r2_ = 2.4675 − 1.2466 × 10^−4^*T* (600 °C < *T* < 1100 °C) (7)

Table 2 shows visualized parameters of the SRR-based RFID temperature sensor we fabricated and other kinds of wireless temperature microwave sensors. As we can see in Table 3, the sensor proposed in this paper has a lower profile and wider temperature sensing range. For the sensitivity and sensing distance of sensors, the resonator-based microwave sensor in Reference [18] has a high sensitivity of 0.24 MHz/°C with a sensing distance of 30 mm, while the dielectric resonance temperature sensor in Reference [6] has a sensitivity of 194 kHz/°C with a sensing distance of about 10 mm. Although the sensor in this paper has a sensitivity of 95.63 kHz/°C which was not so high as the above two sensors, the sensing distance can reach to about 200 mm when using horn antenna.

## 5. Conclusions

This paper presented a novel thin-film sensor based on a metamaterial unit cell using the method of chipless RFID technology. The demand for high-temperature sensing and the applications of the SRR structure have been described. Then, the equivalent circuit diagram of the sensor and the coupling principle with the antenna have been analyzed. The principle of chipless RFID systems has also been described. The simulation and fabrication of the sensor have been presented. The methods for coding the tags were also introduced. Finally, the sensing distance of the sensor was tested and temperature experiments using a muffle furnace were performed. The experimental results showed that the sensor can work within a distance of 20 mm by using a patch antenna and from a farther distance when using horn antenna. Furthermore, when the temperature was increased from 28 to 1100 °C, the frequency of the fabricated sensor decreased from 2.417 to 2.320 GHz with an average sensitivity of 95.63 GHz/°C. Future work will be performed to fabricate SRR-based sensors using 3D printed technology or commercial desktop inkjet printers.

## Figures and Tables

**Table 1:** Parameters of designed sensor.

Parameter	Meaning	Value/mm
R	Radium of substrate	14.0
h	Height of ceramic substrate	0.1
l	Side length of out-ring	13.0
p	Interval between two rings	1.0
t	Width of metal rings	1.0
b	Height of metal rings	35.0 × 10^−3^
s	Width of slot	1.0

**Table 2:** Performance index of alumina ceramics.

Project	Test Condition	Value
Volume density	—	>3.7
Rupture strength	—	3000
Linear expansion coefficient	20–800 °C	6.5–7.5
Dielectric constant	20–100 °C	9–10.5
Loss tangent	20–100 °C	≤2.5

**Table 3:** Parameters of different temperature sensors.

Sensor Type	Profile	Sensitivity	Temperature Sensing Range	Sensing Distance	Working Frequency
Metamaterial Inspired High-Temperature Microwave Sensor	Ø = 13 mm h = 0.1 mm	95.63 kHz/°C	28–1100 °C	200 mm (horn antenna)	Around 2.42 GHz
Dielectric resonance temperature sensor in Reference [6]	29 mm × 29 mm × 5 mm	194 kHz/°C	27–800 °C	About 10 mm	Around 2.44 GHz
Resonator based Microwave sensor in Reference [18]	22 mm × 22 mm × 1.5 mm	0.24 MHz/°C	50–400 °C	30 mm	Around 2.28 GHz
